# SIRT3 Inhibits Cell Proliferation of Nonsmall Cell Lung Carcinoma by Inducing ROS Production

**DOI:** 10.1111/crj.70033

**Published:** 2024-11-05

**Authors:** Ze Yu, Hongtao Liao, Guanhuai Wu, Ying Liu, Guoqiang Zhang, Liang Xiao, Shuibo Yang, Jia Liu, Guocai Yang

**Affiliations:** ^1^ Laboratory of Cytobiology and Molecular Biology, Zhoushan Hospital Zhejiang University School of Medicine Zhoushan Zhejiang China; ^2^ Laboratory of Cytobiology and Molecular Biology, Zhoushan Hospital Wenzhou Medical University Zhoushan Zhejiang China; ^3^ Department of Cardiothoracic Surgery, Zhoushan Hospital Wenzhou Medical University Zhoushan Zhejiang China; ^4^ Department of Pharmacy, Zhoushan Hospital Wenzhou Medical University Zhoushan Zhejiang China; ^5^ Department of General Surgery, Zhoushan Hospital Wenzhou Medical University Zhoushan Zhejiang China; ^6^ Department of Surgery and Oncology Shenzhen Second People's Hospital Shenzhen Guangdong China; ^7^ School of Agriculture SUN Yat‐Sen University Shenzhen Guangdong China; ^8^ Shenzhen Zhongjia Bio‐Medical Technology Co., LTD Shenzhen Guangdong China

**Keywords:** NSCLC, OXPHOS, ROS, SIRT3, tumor suppressor

## Abstract

**Background:**

Sirtuin 3 (SIRT3) is located in the mitochondrial matrix, regulating acetylation levels of metabolic enzymes. As an oncogene or a tumor suppressor gene, SIRT3 plays an important role in the commencement and progression of certain cancers. In this research, we investigated the role of SIRT3 in the progression of nonsmall cell lung carcinoma (NSCLC).

**Methods:**

In this study, bioinformatics was used to analyze the differential expression of SIRT3 in NSCLC tissue and normal tissues, prognosis, single‐cell analysis, and related signaling pathways. The Lentiviral overexpressing SIRT3 was constructed, and CCK8 and colony formation assay were used to evaluate the NSCLC cells proliferation, ROS production was detected by flow cytometry, and the sea‐horse test was used to measure cellular oxygen consumption (OCR).

**Results:**

SIRT3 expression was significantly decreased in NSCLC, and low expression of SIRT3 was closely related to the poor prognosis. Besides, on the whole, upregulation of SIRT3 suppressed cell proliferation in A549 and SK‐MES‐1 cells via increasing oxidative phosphorylation (OXPHOS) and ROS production.

**Conclusions:**

Overall, our findings suggested that SIRT3 functions as a tumor suppressor that can suppress the progression of NSCLC via stimulating ROS production.

## Graphical abstract

NSCLC patient samples can be molecular subtyping according to the expression levels of the SIRT family. SIRT3 is significantly decreased in NSCLC, and low expression of SIRT3 is closely related to poor prognosis. SIRT3 suppressed cell proliferation in NSCLC cells via increasing OXPHOS and ROS production.

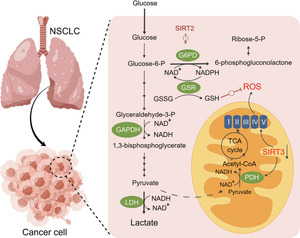

## 1 Introduction

Globally, lung cancer ranks as the most frequently diagnosed cancer and exhibits the highest cancer‐specific mortality rate among both males and females [1]. Annually, around two million fresh instances of lung cancer are detected, leading to 1.7 million new cancer‐related fatalities, accounting for 11.6% of all cancer cases and 18.4% of all cancer‐related deaths worldwide [2]. Nonsmall cell lung cancer prevails as the predominant form of this malignancy [3]. Given the absence of distinct symptoms, early detection of NSCLC remains unattainable, resulting in the diagnosis of a significant majority of patients at an advanced stage [4]. Chemotherapy agents, particularly cisplatin, are considered the preferred therapeutic approach for NSCLC, representing the prevailing choice in clinical practice [5] Despite the continuous development of novel treatment modalities, such as targeted therapy and immunotherapy, the efficacy of therapeutic agents against NSCLC cells has been progressively compromised in recent years, resulting in suboptimal therapeutic outcomes. Consequently, there is an urgent need for comprehensive investigations into the potential biological mechanisms underlying the development of NSCLC, with the aim of devising effective strategies for disease prevention and treatment.

Recently, a proteomic investigation has revealed that approximately 2000 proteins, which play crucial roles in various cellular processes, can undergo acetylation. This finding suggests that inhibiting lysine acetylation holds promise for potential cancer treatment [6, 7, 8]. Furthermore, protein acetylation has been conserved throughout evolution, is responsive to imbalances in intracellular energy levels, and can be regulated in a reversible manner by different deacetylases and acetyltransferases [9].

The Sirtuin family comprises seven proteins (SIRT1–7) that undergo deacetylation by nicotinamide adenine dinucleotide (NAD+). In human cells, SIRT5, SIRT4, and SIRT3 are exclusively localized in mitochondria [10]. SIRT3, functioning as a mitochondrial deacetylase, plays a crucial role in various cellular processes associated with acetylation. Numerous reports have indicated the involvement of SIRT3 in multiple diseases, including certain cancers, diabetes, and cardiovascular disease [11, 12, 13]. Several studies have demonstrated the regulatory role of SIRT3 in mitochondrial function, which has significant implications for fatty acid metabolism, oxidative phosphorylation, and metabolic enzyme activity [14, 15, 16].

However, depending on the genetic background, SIRT3 can either function as an oncogene or as a tumor suppressor gene in different tumors. The objective of this study was to determine whether SIRT3 correlates with NSCLC in clinical samples and cell lines and further explore the molecular mechanism of SIRT3 in promoting or inhibiting the occurrence and development of NSCLC.

## 2 Materials and Methods

### 2.1 Cell Culture

NSCLC cells (A549 and SK‐MES‐1) cultured in DMEM/F‐12 and EMEM medium, supplemented with 10% fetal bovine serum (FBS; Gibco, United States), 1% antibiotics (100 U/mL penicillin and 100 μg/mL streptomycin), respectively. Next, cells were cultured at 37°C, 5% CO_2_. These NSCLC cells were purchased from WuHan Biobank (Wuhan human genetic resources bank).

The Lentiviruses that overexpress SIRT3 were purchased from TsingkeBiotechnology Co., Ltd.

### 2.2 Cell Proliferation

Cell proliferation was examined using the CCK‐8 kit (Beyotime, Shanghai, China) assay according to previously described [17]. A549 and SK‐MES‐1 cells were placed in a 96‐well plate at a density of 5000 cells per well. The cells were infected with lentivirus overexpressing SIRT3, and the positive cells were screened with purinomycin. Cells were incubated in 90% basal culture medium and 10% CCK‐8 solution for 2 h at 37°C. At last, the OD value was measured with a microplate Reader (TECAN, Japan).

### 2.3 Colony Formation

Cells were inoculated into six‐well plates at a density of 200 cells per well. Following a duration of either 15 days, with the medium being replaced every 3 days, the cells were immobilized using 4% paraformaldehyde and subsequently subjected to staining with 0.1% crystal violet (Beyotime, Shanghai, China) for a period of 10 min at ambient temperature. The stained plates were then air‐dried and employed for image capture through microscopic examination at a magnification of 100X.

### 2.4 Western Blot Analysis

Standard procedures were followed in the preparation of protein extracts. A 10% SDS‐PAGE was used to separate the proteins, followed by a transfer to PVDF membranes (Millipore, United States). Then, we incubated the membranes with specific primary antibodies (10099‐1‐AP, SIRT3; 22031‐1‐AP, Vimentin; 55004‐1‐AP, CCNB1; proteintech, China) and Total OXPHOS human antibody Cocktail (ab110411; abcam, United States) at 4°C overnight. ECL detection system was used to detect the levels of SIRT3 protein.

### 2.5 Mito‐ROS Assay

Mito‐ROS production was detected by specific fluorescent probe MitoSOX Red (HY‐D1055, MCE, United States). After treatment, cells were washed and incubated with 10 μM MitoSOX Red for 30 min at room temperature in dark. Then, cells were washed and examined using flow cytometer (BD).

### 2.6 Oxygen Consumption Rate (OCR) Measurements

The Seahorse XF 24 Extracellular Flux Analyzer (Seahorse Bioscience) was utilized to measure OCR. The experiments were conducted in accordance with the instructions provided by the manufacturer of the Seahorse XF Cell Mito Stress Test kit (Agilent Technologies). In brief, A549 and SK‐MES‐1 cells were seeded in a XF 24‐well plate at a density of 1 × 10^4^ per well and were permitted to adhere overnight. Subsequently, the cells were utilized for the assessment of OCR.

Following the initial baseline measurements, a series of injections were administered sequentially, including oligomycin, a reversible inhibitor of oxidative phosphorylation, FCCP, and a combination of rotenone, a mitochondrial complex I inhibitor, and antimycin A, a mitochondrial complex III inhibitor (Rote/AA). The data obtained from these injections were analyzed using Seahorse XF‐24 Wave software. The results were then normalized to the number of cells, and the oxygen consumption rate (OCR) was reported in picomoles per minute.

### 2.7 Bioinformatics Analysis

All Bioinformatics database websites and the methods for bioinformatics analysis were shown in the Supporting Information (supplementary methods).

### 2.8 Energy Metabolism Mass Spectrometry

The detailed methods and conditions for energy metabolism mass spectrometry are presented in the Supporting Information (supplementary materials).

### 2.9 Statistical Analysis

Data are presented as the mean ± SD of three independent experiments. Student's *t* test was applied to determine statistical significance. Statistical significance was set at *p < 0.05, **p < 0.01, and ***p < 0.001.

## 3 Results

### 3.1 Identification of Molecular Subtypes Based on Deacetylase Family

**FIGURE 1:**
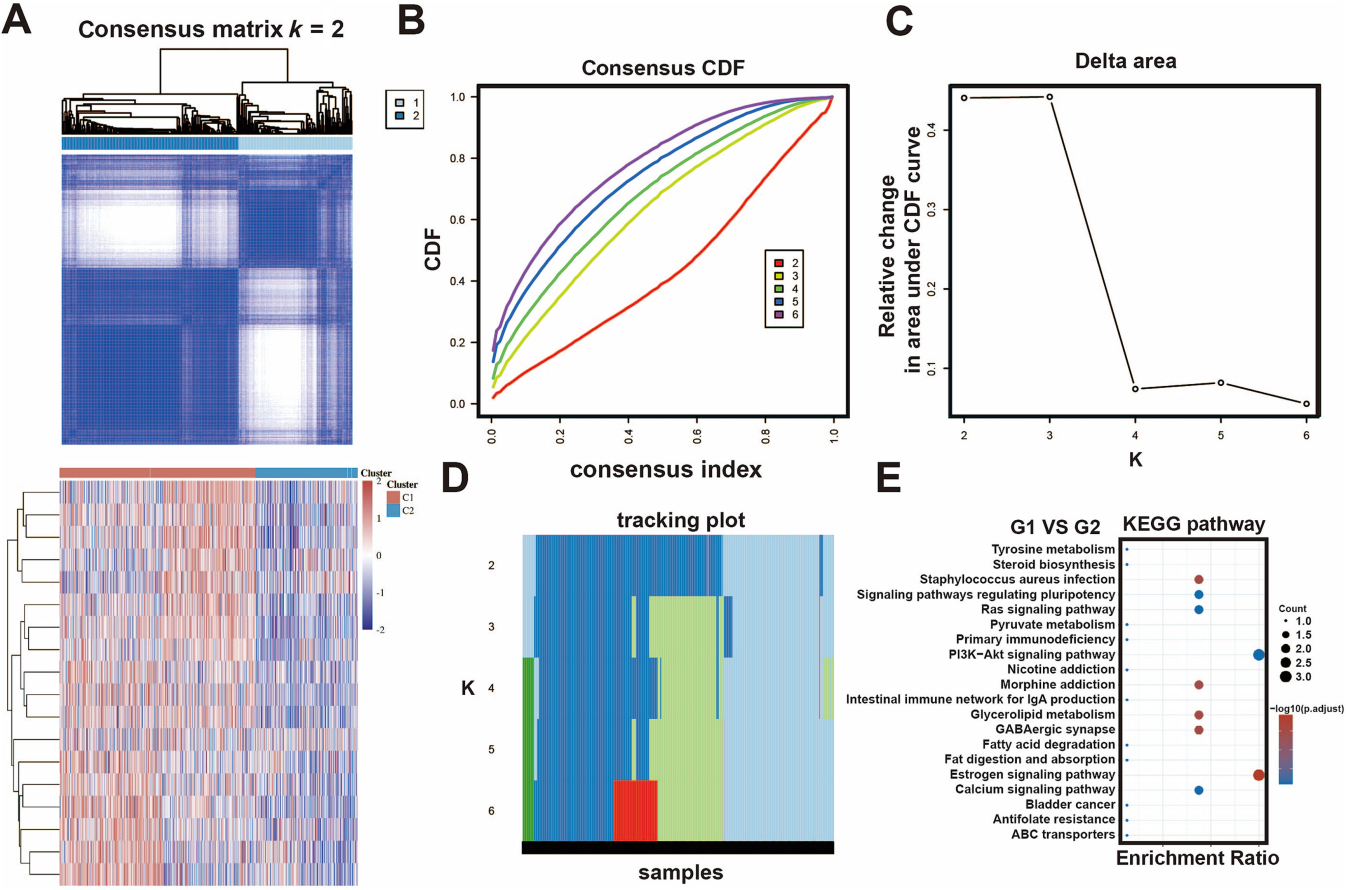
Classification of lung cancer based on HDAC family. (A) Consensus matrix heat‐map for *k* = 2. (B) Consensus clustering CDF for *k* = 2–6. (C) Relative change in area under CDF curve for *k* = 2–6. (D) The tracking plot for *k* = 2–6. (E) The bubble chart of KEGG pathway enriched with DEGs (Cluster 1 vs. Cluster 2).

First, these SIRT family genes were used for NMF cluster analysis, and the comprehensive correlation coefficient was used to determine the optimal *k* = 2. TCGA‐LUAD/LUSC samples were then divided into two different clusters, namely, Clusters 1 and 2. When *k* = 2, the consensus matrix heat‐map had clear boundary and minimal interference between subgroups, indicating that the samples had stable clusters (Figures 1A–D and S1).

In order to better understand the differences between the samples of the two clusters (G1 and G2), we analyzed the differential genes and pathways between the two groups; the results of KEGG pathway enrichment analysis revealed these DEGs were significantly involved to estrogen signal and PI3K‐AKT signal (Figure 1E). These findings revealed that the SIRTs signature could classify NSCLC into distinct molecular subtypes.

### 3.2 Survival Analysis for SIRTs in NSCLC

**FIGURE 2:**
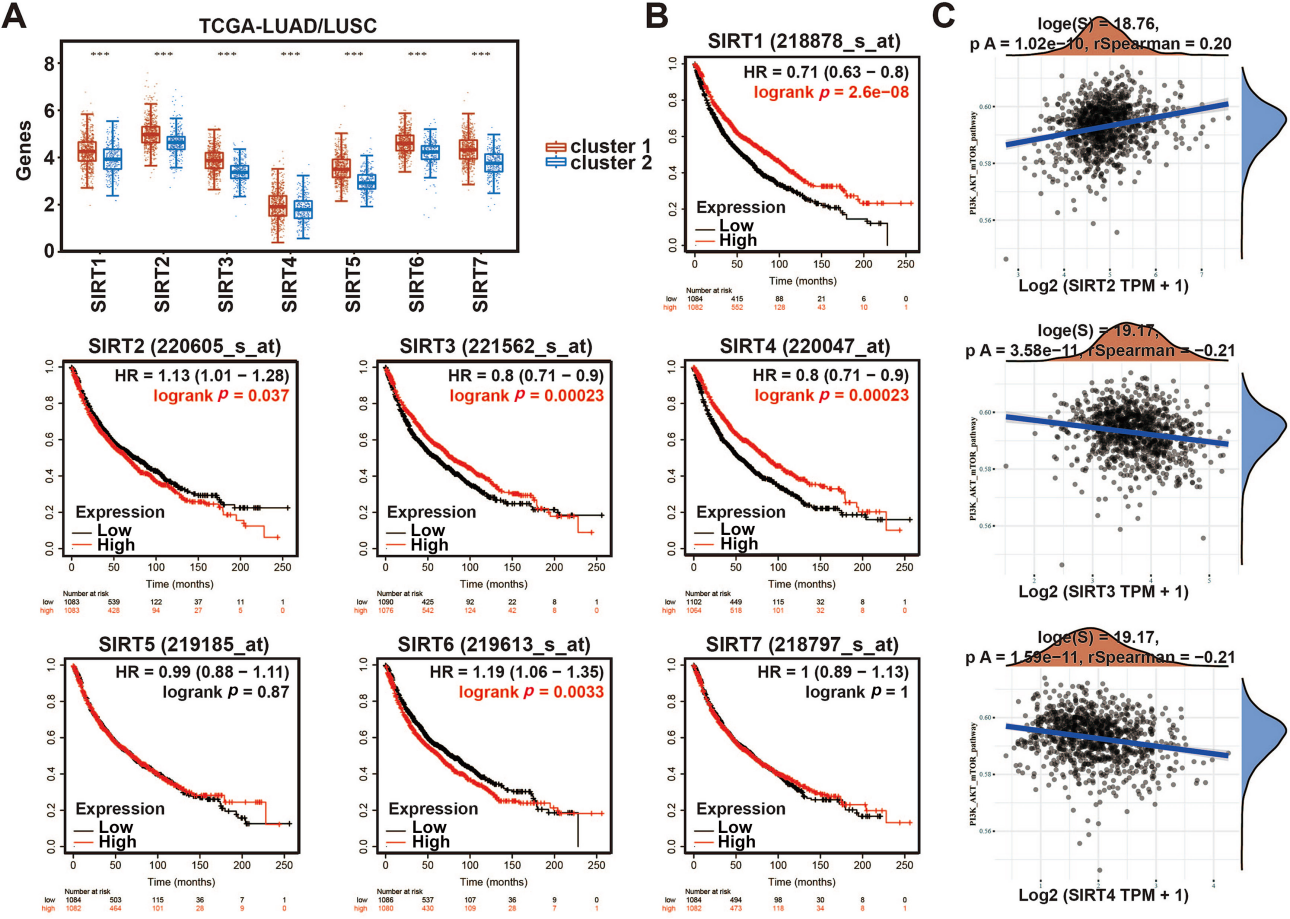
Survival analysis for SIRTs. (A) The expression of SIRT genes in these 2 clusters (B) The survival analysis of SIRT1–7 in Lung cancer (C) Correlation analysis between SIRT2, SIRT3, SIRT4 and PI3K‐AKT signals.

The analysis results of TCGA database showed that SIRT family genes were downregulated in Cluster 2, compared with that in Cluster 1 (Figure 2A). Subsequently, using the K‐M plotter, we investigated the relationship between the expression of SIRTs and prognostic survival rate in lung cancer patients and found that the high expression of SIRT1, SIRT3 and SIRT4 was associated with better prognosis, respectively (Figures 2B and S2A). Besides, we analyzed the possible association between the expression of SIRT3 family members (SIRT1‐7) and clinic‐pathologic feature (pTNM stages: IB and IIB stage) in NSCLC patients. Results displayed that the expression levels of SIRT2, SIRT3, and SIRT7 in IIB stage were significantly lower than that in normal tissues (Figure S2B). Meanwhile, the expression levels of SIRT3 and SIRT4 were significantly negatively correlated with PI3K‐AKT signaling (Figures 2C and S2C). Based on the above analysis, we speculated SIRT3 is highly likely to be involved in the progression of NSCLC as a tumor suppressor. Therefore, next, we tried to excavate the mechanism of SIRT3 through further experiments and bioinformatics analysis.

### 3.3 SIRT3 Is Negatively Correlated With the Proliferation of NSCLC

As we know, SIRT3 localizes to the mitochondria; moreover, interestingly, although the function of SIRT3 in NSCLC has been reported individually, the effects of SIRT3 on lung cancer cells are contradictory in these articles [18, 19, 20, 21]. Therefore, the molecular function of SIRT3 has not been determined.

**FIGURE 3:**
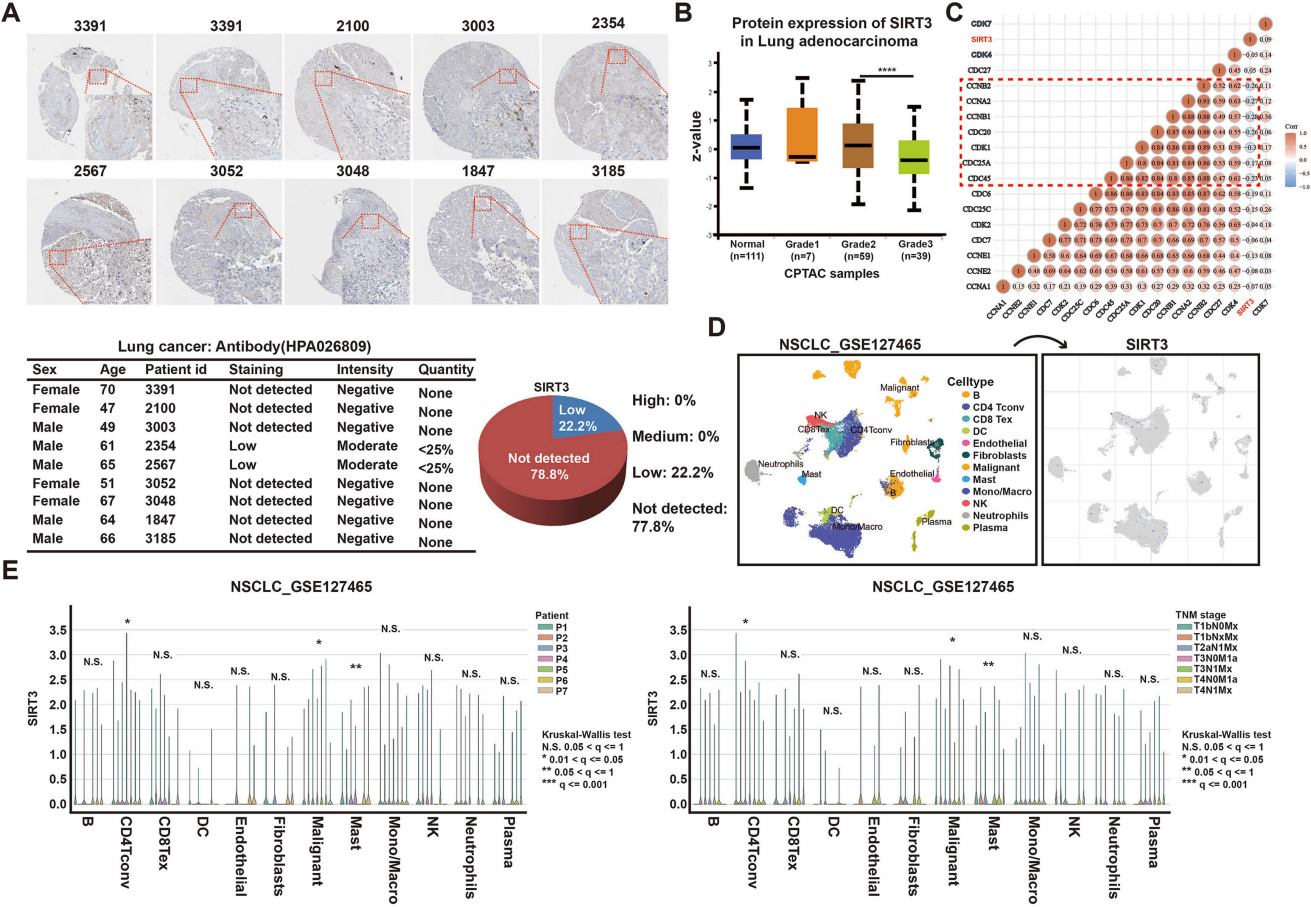
SIRT3 is negatively correlated with cell growth pathways. (A) IHC stain of SIRT3 for NSCLC samples in HPA database (B) The expression of SIRT3 protein at different grades of lung adenocarcinoma tissues (C) Correlations between SIRT3 and cell cycle genes (D, E) Analysis of SIRT3 expression at single cell levels in different NSCLC samples.

First, in order to study the SIRT3 expression in lung cancer samples at the protein level, IHC assay of SIRT3 was analyzed using the Human Protein Atlas (HPA) database, and we selected 10 samples. Figures 3A revealed the staining and intensity of SIRT3 protein in all NSCLC samples. Results displayed that SIRT3 was weakly stained in these samples, indicating SIRT3 was hardly expressed or expressed at very low levels in the tissues of NSCLC patients. Furthermore, we used the Ualcan website to examine the expression of SIRT3 in NSCLC tissues, and the analysis results identified SIRT3 expression was lower in NSCLC with higher tumor histological stage (Figure 3B). Beyond that, we used spearman analysis to explore the correlation between SIRT3 expression and cell cycle marker genes in NSCLC samples; results showed SIRT3 was negatively correlated with major cell cycle marker genes, which also suggests that it has a positive regulatory function on cell proliferation (Figure 3C). Single cell analysis showed SIRT3 was not expressed in most immune infiltrating cells of lung cancer tumor microenvironment, and there was no significant difference expression in different patients and pathological stages (Figure 3D,E).

### 3.4 SIRT3 Suppresses Proliferation of NSCLC Cells

**FIGURE 4:**
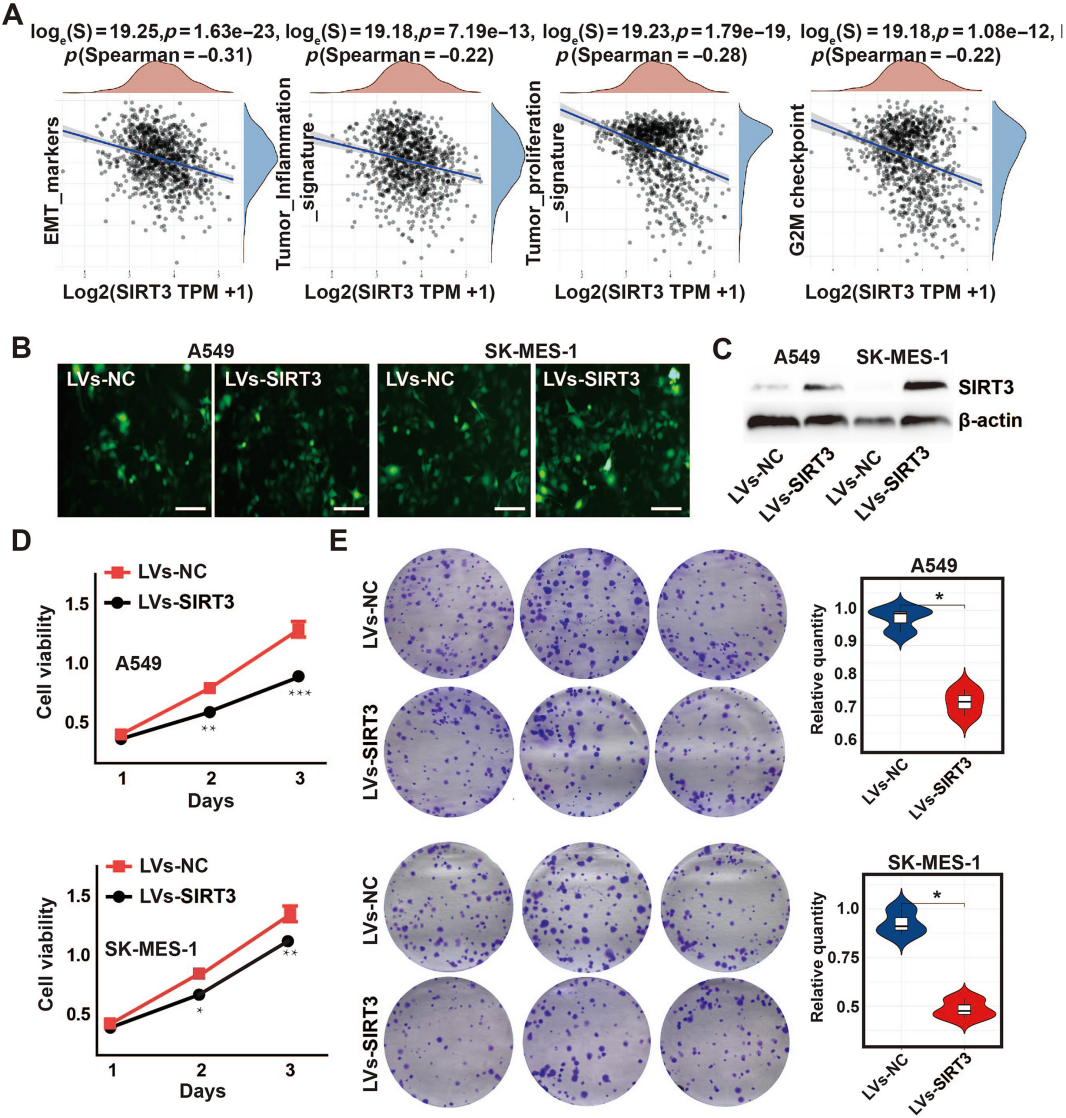
SIRT3 regulates NSCLC cell proliferation. (A) Correlation curve between the SIRT3 expression and main pathways (B) Fluorescence showed the cell infection effect of SIRT3 overexpressing lentivirus (C) The effect of SIRT3 overexpression was analyzed by western blot (D, E) A549 and SK‐MES‐1 cells were treated with overexpressed SIRT3 and then subjected to the CCK‐8 assay and colony formation, respectively. *p < 0.05; **p < 0.01; ***p < 0.001.

At the same time, we analyzed the correlations between individual gene and pathway score using the spearman method and detected a negatively linear correlation between the pathway scores and SIRT3 expression in NSCLC, such as G2M checkpoint and tumor proliferation (Figure 4A). Based on this finding, we detected the marker proteins of EMT and G2/M checkpoints, namely, Vimentin and CCNB1, respectively. And the results showed SIRT3 overexpression could downregulate the protein levels of Vimentin and CCNB1 in A549 cells (Figure S3A). Next, to verify the function of SIRT3 in NSCLC, we constructed a green fluorescently labeled SIRT3 overexpressing lentivirus to infect cells (Figure 4B). Western blot results showed that the virus infection effect was good and SIRT3 was significantly overexpressed (Figure 4C).

Subsequently, we performed CCK8 assays to confirm the influence of SIRT3 on cell viability. Results indicated that SIRT3 overexpression significantly attenuated growth of SK‐MES‐1 and A549 cells, including cell proliferation and clonal formation (Figure 4D,E). Collectively, these data suggested SIRT3 can negatively regulate NSCLC cell growth.

### 3.5 SIRT3 Is Positively Correlated With OXPHOS

**FIGURE 5:**
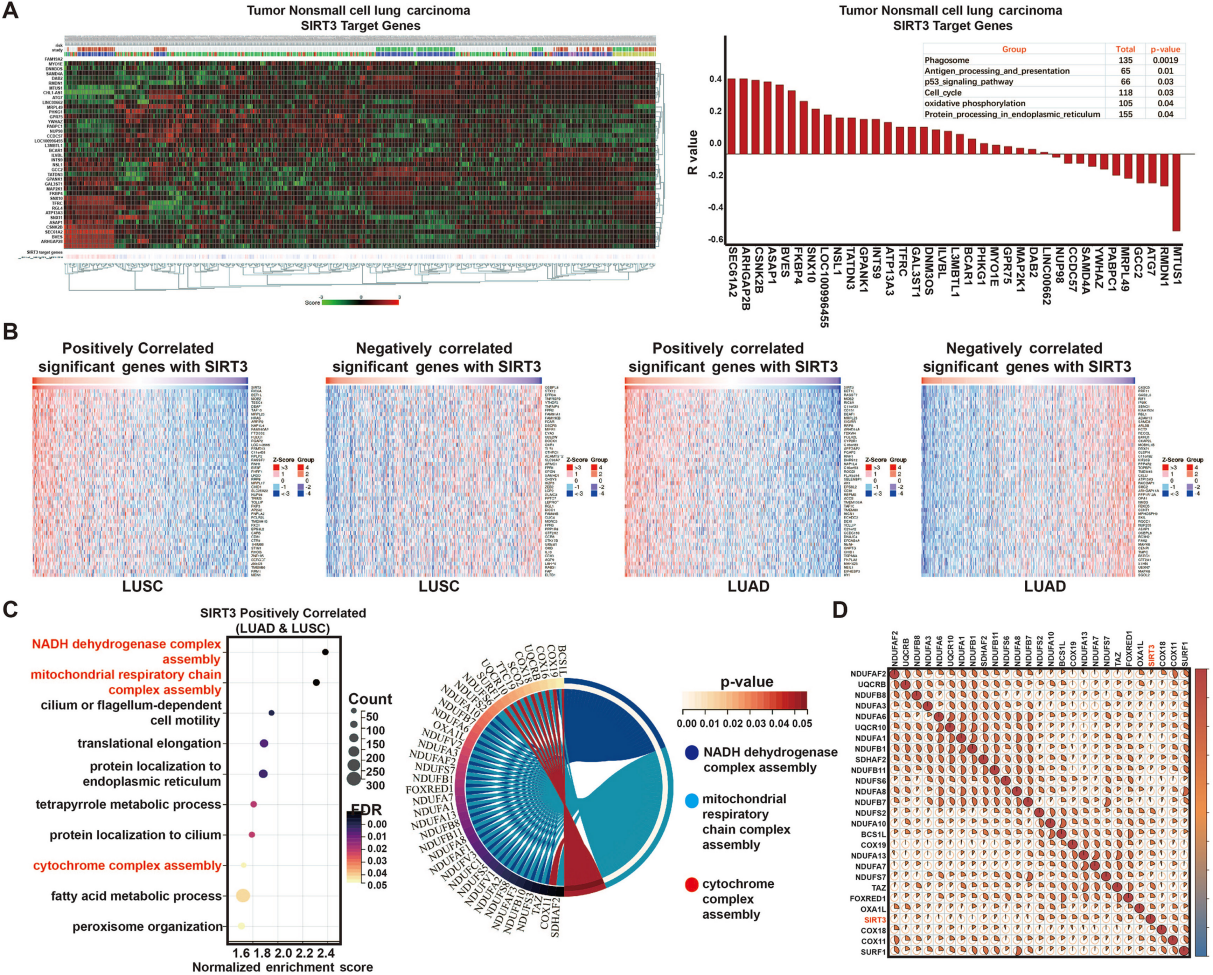
SIRT3 is positively correlated with OXPHOS. (A) SIRT3 target genes in NSCLC (B) Correlated significant genes with SIRT3 in LUAD and LUSC via linkedOmics database (C) Biological Process (GO) term enrichment analysis results of SIRT3 in LUAD and LUSC (D) Correlations between SIRT3 and OXPHOS genes.

To explore the cellular physiological processes involved in SIRT3, pathway enrichment results of SIRT3‐targeted genes showed that these genes were mainly involved in cell cycle, p53 signaling and OXPHOS (Figure 5A).

As well, in order to explore the mechanism by which SIRT3 influences NSCLC, first, we found that the genes and biological processes that positively correlated with SIRT3 expression in both LUAD and LUSC were mainly involved in the mitochondrial respiration chain complex assembly, NADH dehydrogenase complex assembly, and cytochrome complex assembly, which are all key processes of OXPHOS (Figure 5B–D). These data suggested SIRT3, as a mitochondrial protein, may be mainly participated in mitochondrial respiration.

### 3.6 SIRT3 Upregulates the OXPHOS

**FIGURE 6:**
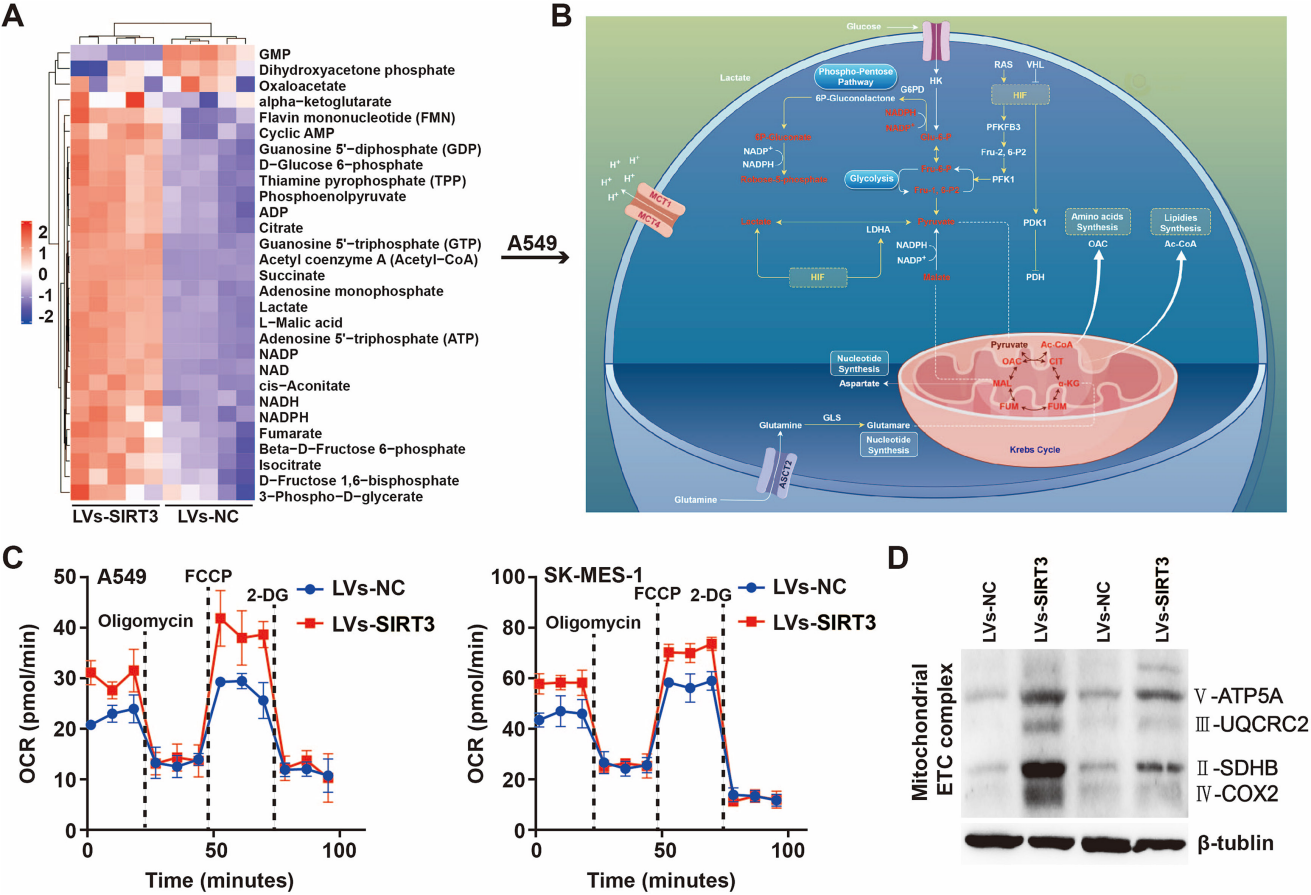
SIRT3 upregulates the OXPHOS.(A) The effects of SIRT3 on glucose metabolism of the NSCLC cells were analyzed through mass spectrometry (B) Schematic illustration of metabolite display of mass spectrometry results (C) OCR upon cells was measured by Seahorse XF in NSCLC cells. Cells were treated with oligomycin, FCCP and Rote/AA for OCR curves. Black dotted line indicated the time point of cell treatment (D) Western blot analysis of total human OXPHOS protein NSCLC cells overexpressed SIRT3 for 48 h.

To investigate the effects of SIRT3 on glucose metabolism in NSCLC cells, we obtained that overexpressed SIRT3 significantly upregulated most metabolites related to oxidized phosphoric acid via energy metabolism mass spectrometry (Figure 6A,B). Additionally, oxygen consumption rate (OCR), which is indicative of OXPHOS, was detected, and the results showed SIRT3 increased the OCR in these two cell lines (Figure 6C). Moreover, SIRT3 significantly increased the levels of OXPHOS‐related proteins ATP5A, SDHB, and COX2 in the western blot analysis of total OXPHOS human antibody cocktail (Figure 6D). Previous energy metabolism mass spectrometry data have shown that overexpression of SIRT3 significantly reduced intracellular ATP (Figures 6A and S3B). Meanwhile, we also utilized the ATP assay‐kit to detect ATP, which was consistent with the mass spectrometry results (Figure S3C).

### 3.7 SIRT3 Increases the ROS Level

**FIGURE 7:**
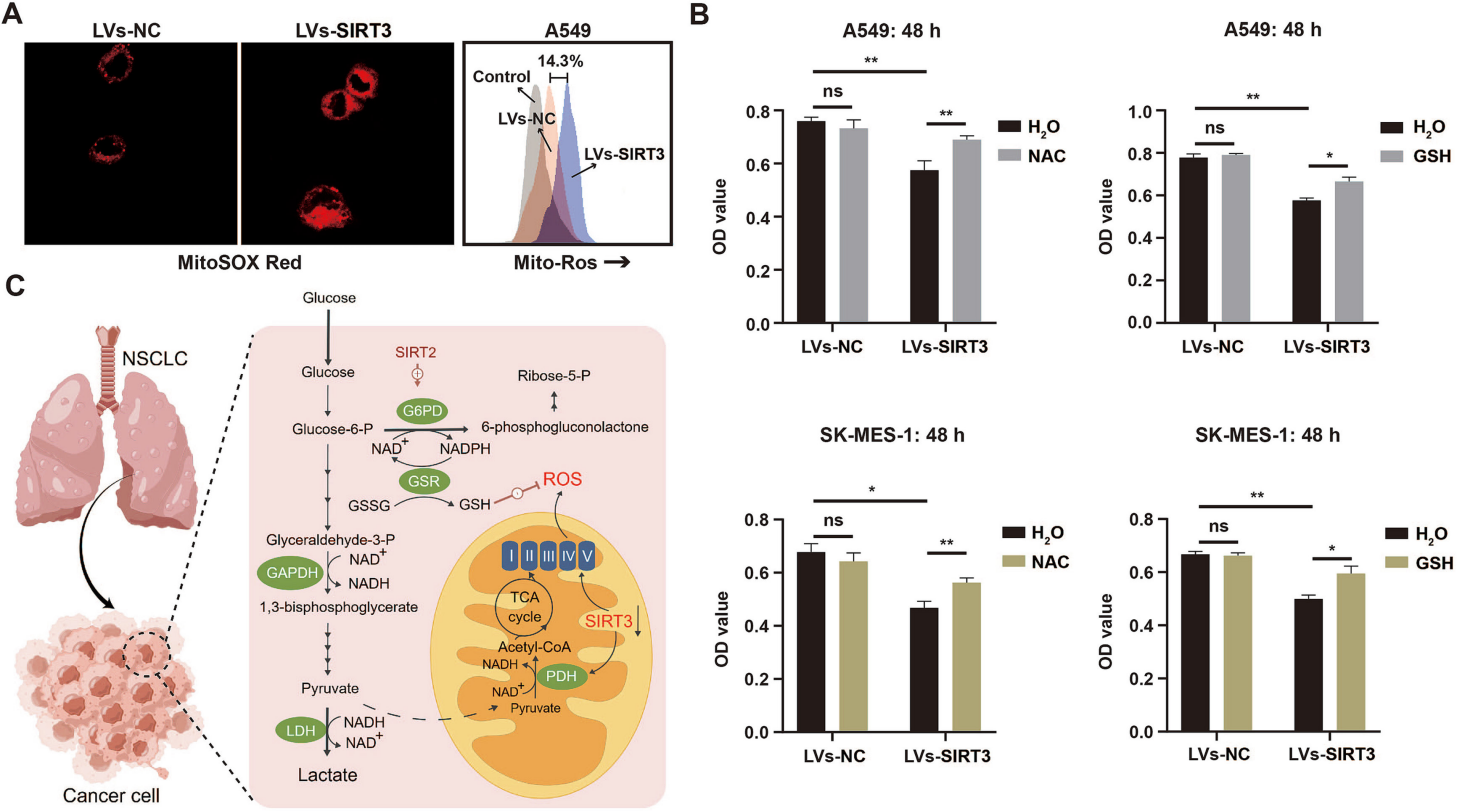
SIRT3 increases ROS in NSCLC cells. (A) Mito‐ROS production was tested by fluorescence and flow cytometry after cells incubated with the fluorescent probe MitoSOX Red. (B) Rescue experiment of antioxidant NAC and GSH restoring SIRT3 induced cell proliferation inhibition (C) The scheme for the role of SIRT3 on the regulation of ROS in NSCLC.

We also estimated the ROS level in NSCLC cells overexpressed SIRT3. These results showed that the ROS was increased with SIRT3 (Figure 7A), and with the addition of the ROS scavenger (NAC or GSH), the suppression of cell proliferation induced by SIRT3 overexpression was also recovered within 48 h (Figure 7B). Therefore, the above findings suggested SIRT3‐induced ROS was an important factor in inhibiting the proliferation of NSCLC cells. Our study has identified a biologic mechanism linking SIRT3 in the regulation of ROS in NSCLC. These results demonstrated SIRT3 could Inhibit NSCLC cell proliferation via increasing intracellular ROS levels (Figure 7C).

## 4 Discussion

Globally, NSCLC continues to be one of the leading causes of death with high rates of occurrence and mortality [1, 2]. Cancer cells exhibit mitochondrial dysfunction [22]. It is known that Warburg effect results in the development of cytosolic aerobic glycolysis as main metabolism for ATP synthesis. As the major mitochondrial deacetylase, SIRT3 activates many oxidative pathways via targeting many enzymes involved in central metabolism. For example, in hepatocytes, SIRT3 induces fatty acid oxidation via deacetylating LCAD [23]. Furthermore, SIRT3 targets the mitochondrial enzymes IDH2 and MnSOD, which contribute to the homeostasis of ROS, and as a result of SIRT3 loss, ROS levels increase, leading to numerous age‐related pathologies, including tumorigenesis [24]. In human cancers, SIRT3 has been found to function as a tumor suppressor by preventing metabolic shifts that promote tumor growth. Downregulation of SIRT3 has been shown to be negatively associated with tumor size, TNM stage, and metastasis in previous studies. There is a possibility that the level of SIRT3 in the serum could serve as a biomarker for noninvasive detection of NSCLC [18].

According to our study, our results show significant decreases in SIRT3 protein and mRNA levels in cancerous tissues compared to normal lung tissues. We propose SIRT3 plays a tumor suppressor role, which is partly due to promote ROS production in NSCLC cells. A number of recent studies have shown that SIRT3 function varies among types of cancer in recent years. For example, SIRT3 suppresses cell proliferation as well as anaerobic glycolysis in breast cancer [25]. Besides, in a variety of cancers, SIRT3 acts as an antioncogene via delaying degradation of p53. Alternatively, other cancers, such as esophageal cancer and oral squamous cell carcinoma, SIRT3 promotes tumor growth [26, 27]. Nevertheless, based on limited research available in the literature, there is no consensus on SIRT3 function in NSCLC [18, 19, 20, 21].

As we know, the immune system has the ability to kill tumors and transformed cells using antitumor immunity. Nevertheless, by a scale for “cancer immune editing,” this ability may be compromised, leading to tumor cells that can evade immune surveillance and trigger tumor growth. In order to survive, the tumor creates an immune‐suppressive microenvironment that would otherwise exist antitumor immune cells can be transformed into dysfunctional, bystander, or even pro‐tumor cells [28]. Therefore, although SIRT3 is negatively correlated with tumor immune infiltration, the effect of immune infiltration on NSCLC cells in this research still needs to be explored in subsequent experiments. Additionally, the effect of SIRT3 on tumor immunity has been little studied. It is reported that SIRT3 inhibited RIPK3‐mediated necroptosis and innate immunity, promoting prostate cancer progression [29]. In addition to triggering TNF‐induced necroptosis, knocking‐down SIRT3 also stimulated macrophage and neutrophil recruitment. In the research of acute lung injury (ALI), SIRT3 has also been demonstrated to play a critical role in the regulation of ROS generation pro‐inflammatory response and macrophage mitochondrial bioenergetics [30]. However, in lung cancer, the role of SIRT3 on immune infiltrate or immune microenvironment has not been studied.

## Author Contributions

Guocai Yang and Ze Yu contributed to the research investigation, writing, and editing. Hongtao Liao and Guanhuai Wu contributed to the in vitro experiment. Ze Yu and Jia Liu completed the bioinformatics analysis. Ying Liu, Guoqiang Zhang, Shuibo Yang, and Liang Xiao contributed to data analysis.

## Conflicts of Interest

The authors declare no conflicts of interest.

## Supporting information


**Figure S1** Consensus matrixes for k = 3 to k = 6 of the 1017 lung cancer patients in the TCGA datasets by clustering the gene expression profile of the SIRT family members.
**Figure S2 (A)**. The survival analysis of SIRTs in Lung cancer. **(B)** The expression of SIRT genes in different tumor p‐TNM stages. **(C)** Correlation analysis between SIRT1, SIRT5, SIRT6, SIRT7 and PI3K‐AKT signals.
**Figure S3 (A)**. The effects of SIRT3 overexpression on Vimentin and CCNB1 were analyzed by western blot. **(B, C)** Effects of SIRT3 on ATP of the NSCLC cells were analyzed through mass spectrometry and ATP test kit.

## Data Availability

For additional inquiries, please contact the corresponding author for the raw data supporting the conclusions in this article.
