# Supplementary material for: SIRT3 Inhibits Cell Proliferation of Nonsmall Cell Lung Carcinoma by Inducing ROS Production
Source: Clin Respir J. 2024 Nov 5;18(11):e70033. doi: 10.1111/crj.70033 (PMC11538276; doi:10.1111/crj.70033)
Supplement: Supplementary file 1 — Figure S1 Consensus matrixes for k = 3 to k = 6 of the 1017 lung cancer patients in the TCGA datasets by clustering the gene expression profile of the SIRT family members. Figure S2 (A). The survival analysis of SIRTs in Lung cancer. (B) The expression of SIRT genes in different tumor p‐TNM stages. (C) Correlation analysis between SIRT1, SIRT5, SIRT6, SIRT7 and PI3K‐AKT signals. Figure S3 (A). The effects of SIRT3 overexpression on Vimentin and CCNB1 were analyzed by western blot. (B, C) Effects of SIRT3 on ATP of the NSCLC cells were analyzed through mass spectrometry and ATP test kit. [file CRJ-18-e70033-s001.docx]

**Supplementary methods**

**Bioinformatics website**

1. RNA-sequencing expression profiles and corresponding clinical information for lung cancer were downloaded from the TCGA dataset. Consistency analysis by using Consensus Cluster plus R package (v1.54.0), the maximum number of clusters is 6, and 80% of the total sample is drawn 100 times, cluster Alg="hc", inner Linkage='ward.D2'. Use the R software (v1.0.12) for clustering heat-maps. The gene expression heat-map retains genes with SD > 0.1. If the number of input genes is more than 1000, it will extract the top 25% genes after sorting the SD. R package were implemented by R version 4.0.3;

The obtained SIRT family genes were used for nonnegative matrix factorization (NMF) clustering. The purpose of NMF was to identify potential features in gene expression profile by decomposing the original matrix into different non-negative matrix. Use the “NMF” R package to perform unsupervised NMF clustering with 1,000 repeat samples and a maximum grouping of 6 on the metadata set. The cumulative distribution function (CDF) and consensus heatmap were used to evaluate the optimal k value, and the TCGA-LUAD/LUSC samples were divided into different clusters according to the expression levels of SIRT family genes.

**Figure 1A**: The abscissa represents category number k, and the ordinate represents the relative change in the area. Consistency of clustering results heat-map (k = 2), Rows and columns represent samples, the different colors represent different types. The expression heat-map of SIRT family genes in different subgroups, **red** represents high expression, and **blue** represents low expression

**Figure 1B, C**: Consensus clustering cumulative distribution function (CDF) and relative change in the area under the CDF curve (CDF Delta area). Relative change in area under the cumulative distribution function (CDF) curves when cluster number varying from k-1 to k.

**Figure 1D**: The black stripe at the bottom of the image represents the sample, showing the classification of sample when k takes different values, and the color blocks of different colors represent different classifications.

**(Figure. 1A-D & Supplementary Figure. 1)**

1. RNA-sequencing expression profiles and corresponding clinical information for lung cancer were downloaded from TCGA. Statistical analyses were performed using R software v4.0.3 (R Foundation for Statistical Computing, Vienna). (**Figure.2A & Supplementary Figure. 2B**)
2. The survival curves were analyzed using data from the Kaplan-Meier plotter website

<http://kmplot.com/analysis/> (**Figure.2B & Supplementary Figure. 2A**)

1. The correlations between individual genes and pathway score were analyzed with Spearman (TCGA) (**Figure.2C & Supplementary Figure. 2C & Figure.4A**)
2. The IHC analysis of SIRT3 were obtained from The Human Protein Atlas:

<https://www.proteinatlas.org/> (**Figure.3A**)

1. Protein expression of SIRT3 in lung cancer based on CPTAC samples were analyzed using data from the ualcan. http://ualcan.path.uab.edu/ (**Figure.3B**)
2. Expression analysis of SIRT3 at the single cell level:

<http://tisch.comp-genomics.org/search-gene/> (**Figure.3D, E**)

1. Analysis of the SIRT3 target genes in NSCLC

https://hgserver1.amc.nl/cgi-bin/r2/main.cgi (**Figure.5A**)

1. Heat-map of significantly correlated genes and KEGG pathways with SIRT3 are derived from LinkedOmics. http://www.linkedomics.org/login.php (**Figure.5B, C**)

**Supplementary Figures and figure legends**

**
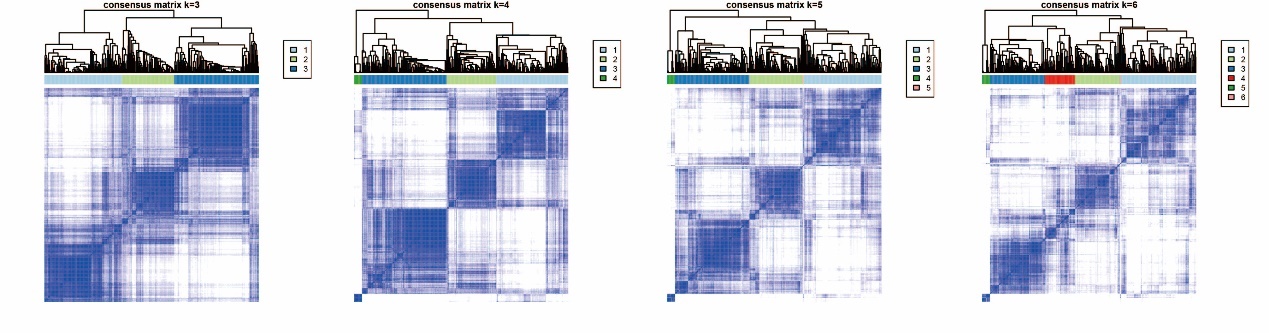
**

**Supplementary Figure 1** Consensus matrixes for k=3 to k=6 of the 1017 lung cancer patients in the TCGA datasets by clustering the gene expression profile of the SIRT family members.


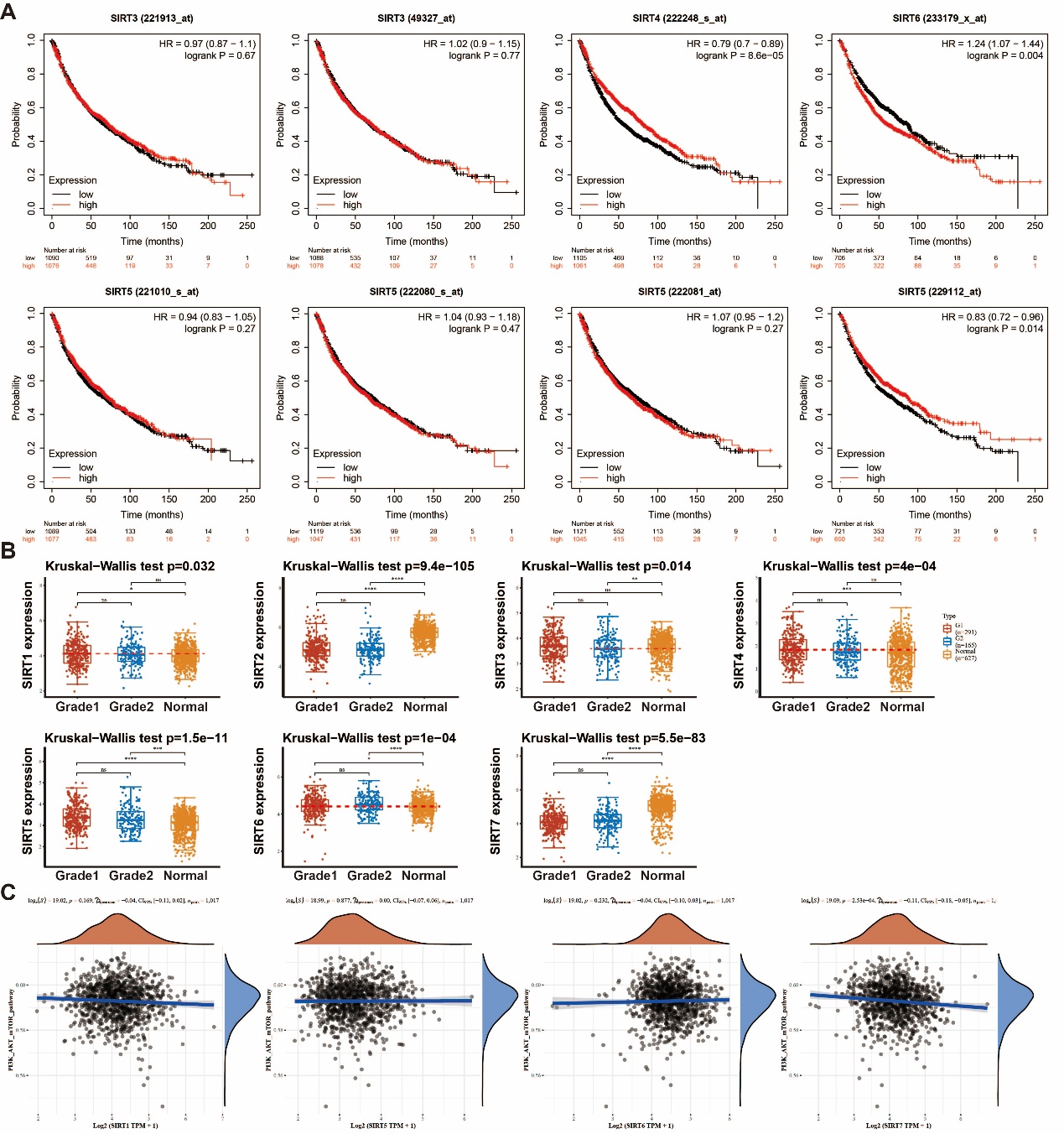


**Supplementary Figure 2 (A)** The survival analysis of SIRTs in Lung cancer. **(B)** The expression of SIRT genes in different tumor p-TNM stages. **(C)** Correlation analysis between SIRT1, SIRT5, SIRT6, SIRT7 and PI3K-AKT signals.


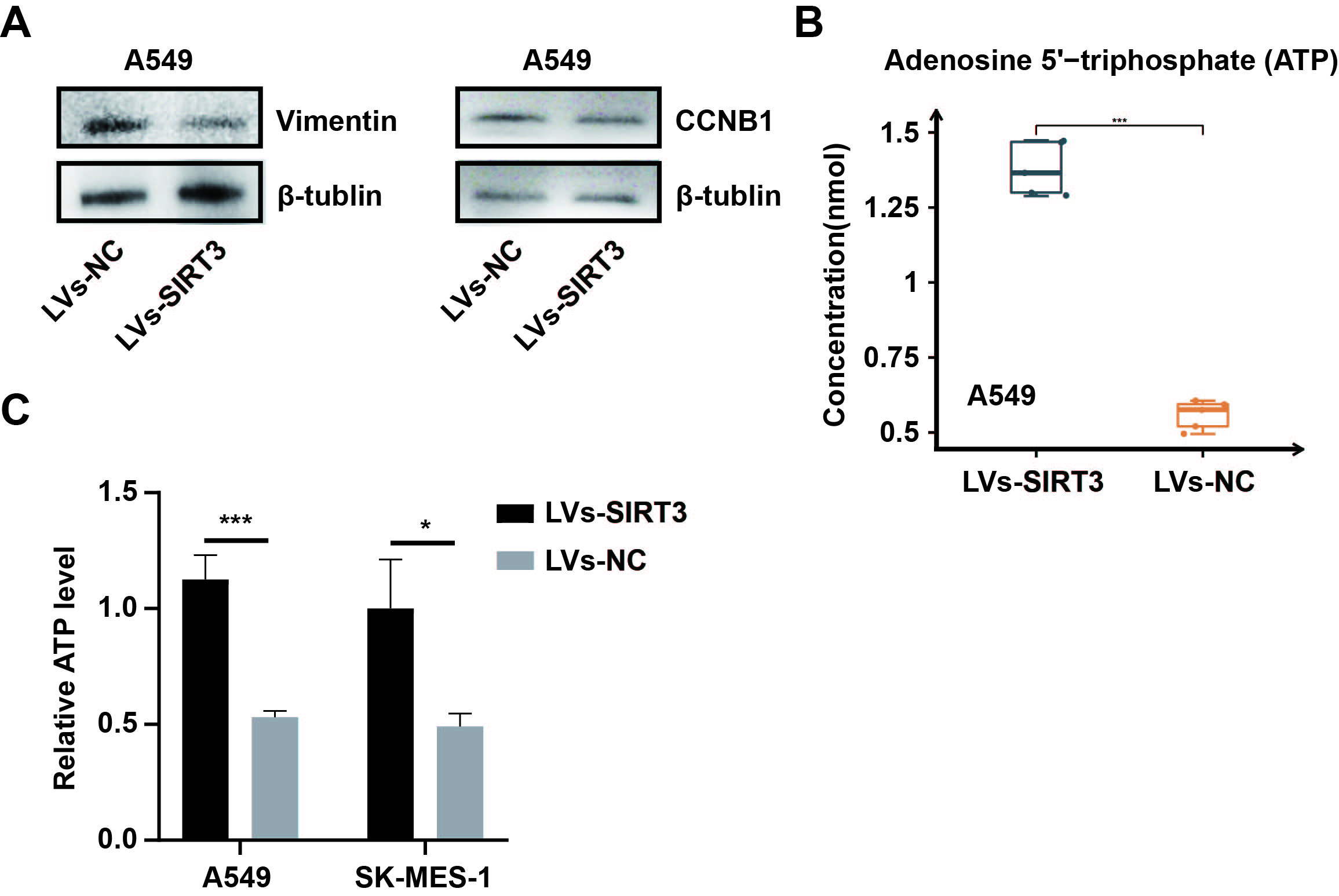


**Supplementary Figure 3 (A)** The effects of SIRT3 overexpression on Vimentin and CCNB1 were analyzed by western blot. **(B, C)** Effects of SIRT3 on ATP of the NSCLC cells were analyzed through mass spectrometry and ATP test kit.

**Supplementary materials and methods**

**1. Metabolite extraction**

Samples were taken out at -80℃, 0.5ml methanol acetonitrile solution (2:2:1, v/v) was added, 10ul (10mMol/L SUCCINIC ACID-D6 internal standard) was added, vortex for 60s, ultrasound was performed at low temperature for 30min twice, and eggs were deposited at -20℃ for 1h White, 14000rcf, centrifuge at 4℃ for 20min, freeze-dry supernatant, store sample at -80℃.

**2. Chromatography-mass spectrometry detection conditions**

2.1 Chromatographic condition

Samples were separated by Agilent Infinity LC ultra-high performance liquid chromatography system. The samples were placed in an automatic injector at 4℃, the column temperature was 35℃, mobile phase A: 50mM ammonium acetate aqueous solution +1.2% ammonium hydroxide, the mobile phase B: 1% acetyl acetone acetonitrile solution, the flow rate was 300 μL/min, and the sample size was 2 μL. The relevant liquid phase gradient is as follows: 0-1min, liquid B 70%, 1-10min, liquid B from 70% linear change to 60%; in 10-12 min, liquid B changed linearly from 60% to 30%. At 12.1-15min, liquid B remained at 30%; at 15-15.5min, liquid B changed linearly from 30% to 70%; at 15.1-22min, liquid B remained at 70%. A QC sample is set up every certain number of experimental samples in the sample queue to detect and evaluate the stability and repeatability of the system. The sample cohort is set to a standard mixture of target substances for correction of chromatographic retention time.

2.2 Mass spectrum condition

A 5500 QTRAP mass spectrometer was used for mass spectrometry in negative ion mode. 5500QTRAP ESI source conditions are as follows: 5500QTRAP ESI Source conditions are as follows: source temperature 450℃, ion Source Gas1 (Gas1): 45, Ion Source Gas2 (Gas2): 45, Curtain gas (CUR): 30, ion Sapary Voltage Floating (ISVF) -4500 V; the ion pair to be measured was detected by MRM mode.

2.3 Data analysis

The chromatographic peak area and retention time were extracted by the Multiquant 3.0.2 software. Retention time was corrected by the standard of the target substance, and metabolite identification was carried out.

**3. ATP detection**

The culture solution was removed, and 200 microliters of lysate were added to each well of the 6-well plate (that is, 1/10 of the amount of 2 ml of cell culture fluid) to lysate the cells. In order to fully lysate the cells, a pipette can be used to repeatedly blow or shake the culture plate to fully contact the lysate and lysate the cells. Normally cells will break immediately upon contact with the lysate. After cracking, centrifuge at 4℃ 12000g for 5 min and take supernatant for subsequent determination.

Add 100 microliters of ATP test solution to the test hole or tube. Leave at room temperature for 3-5 minutes, so that all the background ATP is consumed, thereby reducing the background. It is possible to add 100 microliters of ATP detection working liquid to 10-20 detection holes or tubes at one time, add 20 microliters of sample or standard product to the detection holes or tubes, quickly mix with a gun (micropipette), and determine the RLU value or CPM with a chemical luminometer or liquid flash meter at least 2 seconds after the interval. Finally, the concentration of ATP in the sample is calculated according to the standard curve.
